# Cereblon and IRF4 Variants Affect Risk and Response to Treatment in Multiple Myeloma

**DOI:** 10.1007/s00005-016-0442-6

**Published:** 2017-01-12

**Authors:** Aleksandra Butrym, Piotr Łacina, Justyna Rybka, Monika Chaszczewska-Markowska, Grzegorz Mazur, Katarzyna Bogunia-Kubik

**Affiliations:** 1 Department of Physiology, Wroclaw Medical University, Wroclaw, Poland; 2 Laboratory of Clinical Immunogenetics and Pharmacogenetics, Hirszfeld Institute of Immunology and Experimental Therapy, Polish Academy of Sciences, Wroclaw, Poland; 3 Department of Haematology, Blood Neoplasms and Bone Marrow Transplantation, Wroclaw Medical University, Borowska 213, 50-556 Wroclaw, Poland; 4 Department and Clinic of Internal and Occupational Diseases, Hypertension and Clinical Oncology, Wroclaw Medical University, Wroclaw, Poland

**Keywords:** Cereblon, Interferon regulatory factor 4, Single nucleotide polymorphisms, Multiple myeloma, Disease susceptibility, Stage of the disease, Response to treatment

## Abstract

Multiple myeloma (MM) is a plasma-cell malignancy derived from an early precursor of the B-cell lineage characterised by bone-marrow infiltration, lytic bone lesions, and the presence of a monoclonal protein in serum and/or urine. Interferon regulatory factor 4 (IRF4) is a critical transcriptional regulator in B-cell development and function that is required during immune response for lymphocyte activation and the generation of immunoglobulin-secreting plasma cells. Immunomodulatory drugs, derivatives of thalidomide, are commonly used in therapy against MM. They are known to target a protein called cereblon (CRBN); however, the exact mechanism remains unknown. The present study aimed to assess the association of two (rs12203592 and rs872071) polymorphisms within the *IRF4* gene and two (rs711613 and rs1045433) in the *CRBN* gene with MM susceptibility, progression, and response to treatment. For this purpose, 144 MM patients and 126 healthy individuals were genotyped for the *IRF4* and *CRBN* alleles. The presence of the *IRF4* (rs872071) *G* allele was more frequently detected in patients than healthy individuals (OR 1.78; *P* = 0.034), and this relationship was especially pronounced in women (OR 2.83; *P* = 0.012). The *CRBN* (rs711613) *A* allele-carriers were better responders to the treatment (*P* = 0.012), in particular to thalidomide including therapy (*P* = 0.023). These results underline the prognostic significance of the *IRF4* and *CRBN* polymorphisms in patients with MM.

## Introduction

Multiple myeloma (MM) is a malignancy characterised by the presence of malignant plasma cells in bone marrow, impaired immunoglobulin production, and the presence of a monoclonal protein in serum and/or urine. It is the second most frequent hematological disease and results in severe bone lesions, renal insufficiency, anaemia, and hypercalcaemia (Raab et al. 2009).

Immunomodulatory drugs (IMiDs) are a group of drugs including thalidomide and its derivatives (lenalidomide and pomalidomide) that are commonly used in therapy against MM. Their mechanism of action remained unknown until recently, when cereblon (CRBN) found to be the target of thalidomide (Chang and Stewart. 2011; Huang et al. 2014). Cereblon was originally thought to have a role in cerebral development (hence its name, with -lon standing for the Lon domain) (Higgins et al. 2004). As a target gene of thalidomide, cereblon most likely mediates all known thalidomide effects, such as cell cycle arrest in myeloma cells and teratogenicity in embryos (Ito et al. 2010; Lopez-Girona et al. 2012).

Effects of IMiDs in MM cells are pleiotropic, causing, among others, decrease in expression of anti-apoptotic factor Bcl2, G0/G1 arrest through p21^WAF−1^ up-regulation, and down-regulation of interferon regulatory factor 4 (IRF4) (Lopez-Girona et al. 2012). IRF4 is a member of the IRF family of proteins that is crucial in the development of the immune system. IRF4 plays multiple roles in maturation of blood cells, particularly in the process of B-cell development, including plasma cell differentiation (Klein et al. 2006). IRF4 is required for normal differentiation of B cells into plasma cells (Klein et al. 2006), but it has also been proved to exert major influence on both healthy plasma cells and malignant ones. In myeloma cells, IRF4 is known to up-regulate over 100 genes (as compared to primary plasma cells), and many amongst them are associated with cellular growth and survival, like *MYC*. Because of pleiotropic effects of IRF4 in myeloma cells, multiple myeloma is said to be “addicted” to IRF4, with lower expression thereof severely hurting myeloma cells (Shaffer et al. 2008).

The aim of this study was to assess relationship of four selected single nucleotide polymorphisms (SNPs) in *IRF4* and *CRBN* genes with risk for disease, response to treatment and other prognostic factors in MM. It is a continuation of our previous study, where we investigated two SNPs in the gene coding for β-catenin and one (rs121918368C > T) within the cereblon encoding gene (Butrym et al. 2015).

The latter one was located within the coding region but we did not observe the presence of its polymorphic variant in our Polish (MM patients and healthy controls) population. Currently, two further SNPs within the *CRBN* gene were selected that may potentially be involved in the control of the *CRBN* gene expression. Our previous studies showed that substitutions within the untranslated regions (UTR) may be associated with the disease (Butrym et al. 2015). Therefore, the substitutions chosen, rs711613A > G and rs1045433A > G, are located in non-coding regions (intron 1 and 3′UTR, respectively) of the *CRBN* gene, itself located in chromosome 3, at the location 3p26.2. They were never studied before and any effects of those variants on cereblon are unknown.

The IRF4 encoding gene is located in the short arm of chromosome 6, at 6p23-p25 (Do et al. 2010; Grossman et al. 1996). Two known SNPs of this gene, rs12203592 and rs872071, were subject of this study. rs12203592 is a C > T SNP located in intron 4 and known to affect pigmentation; it is considered as a risk factor in skin cancers (Han et al. 2011). Its *T*-variant is known to be associated with lower expression levels of the *IRF4* gene (Han et al. 2011), which means that it could potentially affect not only pigmentation-related diseases, but also other ones, such as multiple myeloma.

The other SNP investigated in this study is rs872071. It is a A > G substitution located in the 3′UTR. It has been shown earlier that this SNP was a risk factor of two haematological diseases, chronic lymphocytic leukemia and Hodgkin lymphoma. In both cases, the *G* allele was more common in patients than in healthy controls (Broderick et al. 2009; Di Bernardo et al. 2008). It was also reported that the *G* allele is associated with lower expression of IRF4 (Di Bernardo et al. 2008). Association of the rs872071 polymorphism with risk for multiple myeloma has been investigated in a study on a group of British Caucasian patients, but no such association was found (Pratt et al. 2010).

## Materials and Methods

### Patients and Controls

For this study, we used a group of 144 Polish MM patients who were already studied in our previous work about *CRBN* and *CTNNB1* SNPs. Detailed information available in manuscript of Butrym et al. (2015). In addition, 126 healthy individuals of both sexes served as a control group for the *IRF4* SNPs, while 237 healthy individuals served as a control group for the *CRBN* SNPs.

### Genotyping

DNA was extracted from samples of peripheral blood taken on EDTA using Maxwell 16 Blood DNA Purification Kit (Promega Corp., USA) or silica membranes (Qiagen, Germany) following the recommendations of the manufacturers. Determination of the *IRF4* and *CRBN* polymorphisms was carried out by the LightSNiP typing assay (TIB-MolBiol, Berlin, Germany) on a LightCycler 480 Real-Time PCR system (Roche Applied Science, Mannheim, Germany). Amplifications were performed following the recommendation of the manufacturer.

### Statistical Analysis

Fisher’s exact test was used to test the null hypothesis that there is no difference between allele and genotype frequencies between patients and controls. The odd’s ratio (OR) was calculated by Haldane’s modification of Woolf’s method. In addition, the Statistical Package for Social Scientists (SPSS, SYSTAT 10) was used for multivariate logistic regression analysis. Probability values <0.05 were considered statistically significant, and those between 0.05 and 0.10 as indicative of a trend. All genotypes were tested for deviations from Hardy–Weinberg equilibrium using the *χ* ^2^ test.

## Results

### *CRBN* Polymorphisms and Response to Treatment with Thalidomide

Of the 144 patients genotyped, we had data about response to treatment for 131 individuals. The response rate was 107/131, with the rest of patients progressing or having stable disease after treatment.

Analysis of gene and allele frequencies showed no statistically significant differences between patients and controls in the case of either of the two *CRBN* polymorphisms. We observed, however, major differences regarding response to treatment. The rs711613 *A* allele was more common in patients who had a complete or partial remission after first-line therapy as compared to patients in whom the disease either remained stable or progressed (*P* = 0.012). Multivariate analyses were employed to assess the independent association of the *CRBN* gene polymorphism with the treatment outcome. Age of patients, sex and stage of the disease assessed according to the Duire-Salomon criteria and International Staging System (ISS) score were also considered in these analyses. The analyses confirmed the effect of the rs711613 *A* variant as a protective factor associated with better response to treatment (OR 0.306, 95% CI 0.125–0.753; *P* = 0.010), while more advanced disease (ISS score: 3) appeared to be a risk factor of unfavourable prognosis (OR 4.100, 95% CI 1.586–10.598; *P* = 0.004). Similar relationships were observed when ISS score >II was considered (individual data not shown).

The association of the *CRBN* rs711613 *A* allele was also seen in a group of patients who were treated with thalidomide, alone or in combination with other drugs (*P* = 0.023), but was not statistically significant in a subgroup of patients treated with cyclophosphamide–thalidomide–dexamethasone (CTD; *P* = 0.086).

We also noticed that rs1045433 *G* allele tended to be more common in patients with complete or partial response after any first-line therapy and therapy with first-line therapy involving thalidomide, but those two associations were not statistically significant (*P* = 0.081 and *P* = 0.092, respectively).

Furthermore, we have also found that the rs711613 *G* allele is much more common in patients in stages I–II (according to the Durie–Salmon criteria) than patients who are in stage III (*P* = 0.005).

### Associations of *IRF4* rs872071 with Disease Susceptibility and Progression

The comparison of gene and allele frequencies between controls and cases showed that the rs872071 *G* allele was more frequent in cases compared with controls (0.504 and 0.397), with odds ratio 1.73 (95% CI 1.02–2.94; *P* = 0.046). Stratifying cases and controls according to gender revealed that the increase of the *G* allele in cases compared with controls was even more pronounced in women, with the OR 2.73 (95% CI 1.24**–**6.01; *P* = 0.013), while no statistically significant association was observed in men (OR 1.17, 95% CI 0.57–2.41; *P* = 0.714). Moreover, the *GG* genotype alone was also found to be more common in cases (24.6%) compared with controls (9.5%). The odds ratio for this association was 3.11 (95% CI 1.14**–**8.47; *P* = 0.037).

In addition, an association with progression of the disease was observed. The rs872071 *G* allele was more common in MM patients in stages I–II (according to Durie–Salmon staging system) compared with patients in the final stage III: among the patients in stages I–II 80.8% carried the *G* allele, while only 63.3% in stage III (OR 2.44, 95% CI 1.09–5.48; *P* = 0.038). Adjusting for the duration of disease resulted in a slightly different odds ratio (OR_adj_ 2.30, 95% CI 1.01–5.24).

Stratifying all the patients into two groups according to the duration of disease revealed that the rs872071 *G* allele was more commonly found in patients suffering from MM longer than 3 years compared with patients suffering there from for 3 years or shorter. In the former group, 85.4% were carriers of the *G* allele, while in the latter, only 68.7% (OR 2.67, 95% CI 1.06–6.74; *P* = 0.038). The odds ratio and 95% confidence intervals changed after adjusting for age: OR 2.54, 95% CI 0.98–6.59.

### Lack of Significant Relationships with the rs12203592 SNP

No statistically significant associations with either risk, progression or duration of disease were observed with the rs12203592 polymorphism. Furthermore, no association with response to treatment was found in either of the two studied SNPs.

### Detailed Information on Genotypes

Detailed data for genotypes of all four SNPs in both patients and controls are presented in Table 1 and will be published in MultiGenBank—the immunogenetic database of the Polish population accessible under http://multigenbank.pl/en.

**Table 1:** Genotype frequencies for the polymorphisms under study

Gene	Polymorphism		Genotypes		
			AA (%)	AG (%)	GG (%)
*CRBN*	rs711613A > G	MM	11.0	46.7	42.3
		Controls	11.5	46.6	41.9
	rs1045433A > G	MM	66.4	30.7	2.9
		Controls	64.2	31.5	4.3
*IRF4*	rs872071A > G	MM	25.6	48.2	26.3*
		Controls	37.3	46.0	16.7*

Gene	Polymorphism		Genotypes		
			CC (%)	CT (%)	TT (%)
*IRF4*	rs12203592C > T	MM	83.2	16.8	0.0
		Controls	87.3	12.7	0.0

* This SNP is a risk factor in a recessive model, *OR* 1.73, *P* = 0.046 (*P* = 0.013 in women only)

## Discussion

In our study, we demonstrated that the two SNPs in the non-coding regions of the *CRBN* gene, rs711613 and rs1045433, are associated with different response to treatment in general and to treatment with thalidomide in particular. As the SNPs in question do not result in different protein structure, it is possible that they exert their influence by modulating expression of cereblon. It has already been shown that cereblon levels do influence outcome of IMiD-based therapy and patients with lenalidomide resistance have decreased levels of it. In addition, CRBN expression was reported to not affect response to other anti-myeloma agents, which may explain why we found no association between the two *CRBN* polymorphisms and response to CTD therapy (including thalidomide and two other agents) (Schuster et al. 2014).

Regarding *IRF4*, the SNP we have investigated, rs872071 A > G, is located in the 3′UTR of the gene and has been associated with increased risk for chronic lymphocytic leukemia and Hodgkin lymphoma, suggesting that it might also be a risk factor in other haematological diseases. It affects expression levels of IRF4, as the *G* variant has been shown by Di Bernardo et al. to decrease levels of IRF4 mRNA (Di Bernardo et al. 2008; Broderick et al. 2009). Earlier study by Pratt et al. (2010) supplied no evidence for an association between this SNP and risk for multiple myeloma. No association was observed in both adjusted for sex and age and unadjusted analysis. It should also be noted that that study used a larger number of patients than ours, but nevertheless, they noted that their failure to show an association with MM might have been due to their study not having been sufficiently powered (Pratt et al. 2010). Interestingly though, the study by Pratt indicated a female-only association with hypercalcaemia, one of the major organ dysfunctions in MM (Pratt et al. 2010).

Our previous studies in MM patients showed a favourable effect of the *CXCL12*-*3′A* variant (rs1801157) with respect to the progression of the disease (less advanced stage) and survival (Mazur et al. 2013) and confirmed the lack of correlations between IL-6 (rs1800795, −174 G > C) and IL-10 (rs1800896, −1082 G > A) SNPs and susceptibility to MM (Mazur et al. 2005).

**Fig. 1:**
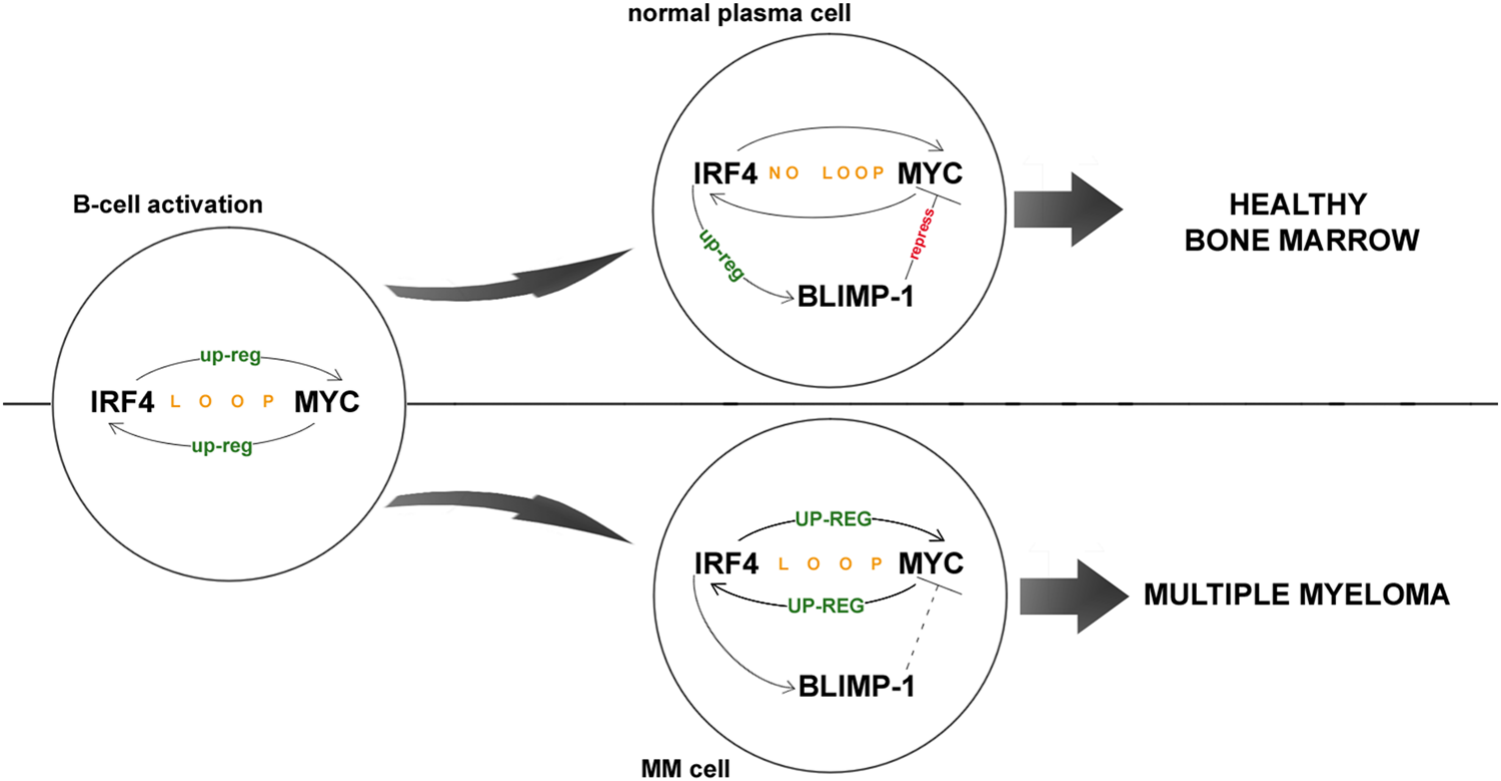
Role of IRF4 expression in myeloma cells. *IRF4* and *MYC* form an auto-regulatory loop, with the former up-regulating the latter and vice versa. This loop is required during the B-cell activation, but is later abrogated in mature plasma cells through expression of *BLIMP*-*1*, which represses *MYC*. In myeloma cells, this mechanism of repression is somehow inactivated, causing the *IRF4*-*MYC* loop to spiral of control, facilitating tumor growth. Increased levels of *IRF4* (as in people with rs872071G allele) could impede the *IRF4*-*MYC* loop, thus slowing down the progression of the disease. On the other hand though, increased levels of *IRF4* would result in lower levels of BLIMP-1, which could help establish the *IRF4*-*MYC* loop during plasma cell formation, thus helping in tumorigenesis

Our present study revealed that the *G* allele of rs872071 does indeed constitute a risk factor in multiple myeloma, which is in line with similar findings in other hematological diseases. The association was significant in women (*P* = 0.012) and not in men (*P* = 0.716), which makes it consistent with previous report by Pratt et al. (2010) that the *G* allele is a female-only risk factor for hypercalcaemia in MM. However, the molecular mechanisms behind this association are not clear. Di Bernardo et al. (2008) have hypothesised that in CLL, the causal allelic variant increases risk by arresting the IRF4-dependent transition of memory B cells into plasma cells. However, because myeloma cells are derived from plasma cells, this model would suggest a different result than the one we and Pratt et al. (2010) observed, i.e., lower IRF4 levels in people with *G* allele would decrease the number of plasma cells and thus lower the risk for multiple myeloma, instead of increasing it. Because of this, we concluded that there must be other factor(s) involved that we did not consider in our study. We conducted a thorough research of the available literature related to IRF4 and based thereon, we propose a different model for the rs872071 *G* as a risk factor in MM, based on the interaction of IRF4 with the transcriptional repressor B lymphocyte-induced maturation protein 1 (BLIMP-1). BLIMP-1 is up-regulated by IRF4 and is known to repress expression of many genes associated with growth in B cells, like *MYC* (Sciammas et al. 2006; Shaffer et al. 2008). It is expressed in plasma cells to stop *MYC* expression, which is necessary during B-cell activation. This mechanism is somehow abrogated in myeloma cells. As a result, *MYC* is overexpressed following the translocations of its gene, which in turn induces overexpression of IRF4, thus forming a loop which helps the myeloma cells survive and grow (Shaffer et al. 2008). Reduced expression of IRF4 in people carrying the rs872071 *G* allele should lead to reduced expression of BLIMP-1, which, given the function of BLIMP-1 as a *MYC* repressor, could help in abnormal growth of *MYC* expression during the formation of multiple myeloma (Fig. 1).

Furthermore, we have also found that the rs872071 *G* allele is more frequent in MM patients in stages I–II of the disease than in patients in stage III (according to Durie-Salmon staging system), and is thus associated with slower progression of disease. This association was still observed after the results had been adjusted for duration of disease. These findings are in line with previous observations showing that mature myeloma cells are “addicted” to IRF4, which controls expression of a host of different genes associated with growth and survival, among them *STAG2*, *CDK6,* and *MYC* (Shaffer et al. 2008). Lower expression of *IRF4* in people the allele apparently results in lower expression of all its target genes necessary for tumor growth, thus slowing its progression down.

We have also ruled out the rs12203592 SNP as a potential risk factor for multiple myeloma. It is a known risk factor for skin cancer and has been known to influence *IRF4* expression (Do et al. 2010). It is possible that the effects of this polymorphism occur in conjunction with unknown tissue-specific transcriptional factors.

In summary, our present study contributes to the reports on genetic factors in MM. Obviously, these observations warrant further, more extended study.
